# Seafood supply mapping reveals production and consumption mismatches and large dietary nutrient losses through exports in the United Kingdom

**DOI:** 10.1038/s43016-024-01102-x

**Published:** 2025-01-02

**Authors:** Anneli Löfstedt, Bernhard Scheliga, Magaly Aceves-Martins, Baukje de Roos

**Affiliations:** 1 The Rowett Institute, University of Aberdeen, Aberdeen, UK; 2 Digital Research Services, University of Aberdeen, Aberdeen, UK

**Keywords:** Sustainability, Agriculture

## Abstract

Seafood can contribute towards healthy and sustainable food systems by improving public health and helping achieve net zero carbon emissions. Here, we provide a high-resolution perspective on UK seafood supplies and nutrient flows at the species level. We mapped seafood production (capture and aquaculture), trade (imports and exports), purchases (within and out of home) and seafood consumption between 2009 and 2020. UK dietary recommendations for finfish consumption were not achieved by domestic production nor national supplies. Mapping dietary nutrient flows revealed that the UK undergoes substantial losses of omega-3 fatty acids, vitamin B_12_ and vitamin D, which could contribute 73%, 46% and 7% towards UK-recommended nutrient intakes, respectively, through exports of oily fish such as salmon, herring and mackerel. Policies should consider promoting greater consumption of locally produced oily fish species to improve public health and seafood system resilience.

## Abstract (web summary)

A mapping exercise reveals that seafood exports from the UK lead to substantial losses of important nutrients. Finfish consumption recommendations cannot be met by domestic production nor national supplies.

## Main

Food is the single strongest lever for optimizing human health and environmental sustainability. Dietary risks are a leading cause of non-communicable diseases and between 20% and 25% of adult deaths are associated with poor diets^1^. At the same time, our diets and the associated global food system contribute a third of global greenhouse gas (GHG) emissions^2^. Transforming diets is essential to alleviate the global health burden, and building a more sustainable food system is paramount to achieving net zero by 2050. Proposals for healthier diets associated with a reduction in GHG emissions include adopting plant-rich diets, that is, those rich in vegetables, and increasing the consumption of oily fish, as recommended in diets such as the Mediterranean or planetary health diet^3^. Fish consumption is associated with a reduced risk of mortality of coronary heart disease^4^. Furthermore, most seafoods provide more nutrients than land-based protein alternatives, at lower emissions^5^. In fact, studies modelling for optimal sustainable diets (that is, those with reduced GHG emissions) found that fish was the most common item to be added to diets to make them healthier and more sustainable^6^. Therefore, it is probable that seafood will feature more frequently in global diets as we transition to net zero. The EAT-Lancet commission recommends consumption of 28 g (range 0–100 g) of fish or shellfish per day, equivalent to 1–2 servings per week, as part of a healthy net zero diet^3^. However, ensuring sustainable seafood supplies that satisfy demand, meet national recommendations for fish consumption and, at the same time, contribute to reference intakes of relevant nutrients requires a better understanding of seafood supplies and nutrient flows at the national and international levels.

Given the international landscape in the trade of fish and fishery products, seafood supply chains are notoriously complex. Global trade and foreign fishing considerably contribute to the redistribution of fish from location of capture^7^ and of marine nutrient supplies^8^. It lessens the restrictions of geographical location, providing countries access to larger quantities and a greater diversity of aquatic foods. On the nutrient level, many countries experience net gains in seafood-relevant nutrients, both through fish imports and foreign fishing^8^. However, fishing patterns and fish distribution result in country-specific profiles for national fish supplies, with high-income countries driving international trade patterns based on economic value rather than nutrient supply, leading to trade imbalances and inequitable distribution of nutrients^8^. High-income countries with mostly affluent consumers often rely more on imports, where the supply chain allows the transportation of aquatic products^9^. For example, in the USA, a third of all aquatic foods consumed in 1961 were imported, which rose to nearly three-quarters in 2019^10^. In the UK, domestic production supplied almost 90% of UK consumer demand up to 1975, whereas currently between 60% and 80% of consumed seafood is imported^11^. At the same time, the UK experiences net losses in seafood-relevant nutrients through exports^8^.

Transformation of national food systems, particularly seafood systems, requires nutrient-sensitive policies that optimize both food and nutrient security, based on high-resolution and context-specific data, linking production, trade and diets^12^. We mapped seafood supplies in the UK, by species and sector, as a case study for high-income countries worldwide that have a high reliance on trade to keep fisheries and aquaculture profitable but also to meet consumer demand and preferences^13^. We aimed to obtain an integrated and cross-sectoral understanding of how seafood consumption in the UK relates to food supply chains—what is being produced, what is imported and exported, and how this relates to what we eat and what we should be eating for optimal health. This knowledge will allow a better alignment of fisheries and public health policies and help to reconsider seafood supply chains in terms of food and nutrient supply. It will also ensure that current UK national dietary guidelines consider seafood supplies and the sustainability of production systems, rather than focussing on consumer health outcomes, as is currently the case.

## Results

### Mapping production, trade, purchase and consumption data between 2009 and 2020

**Fig. 1:**
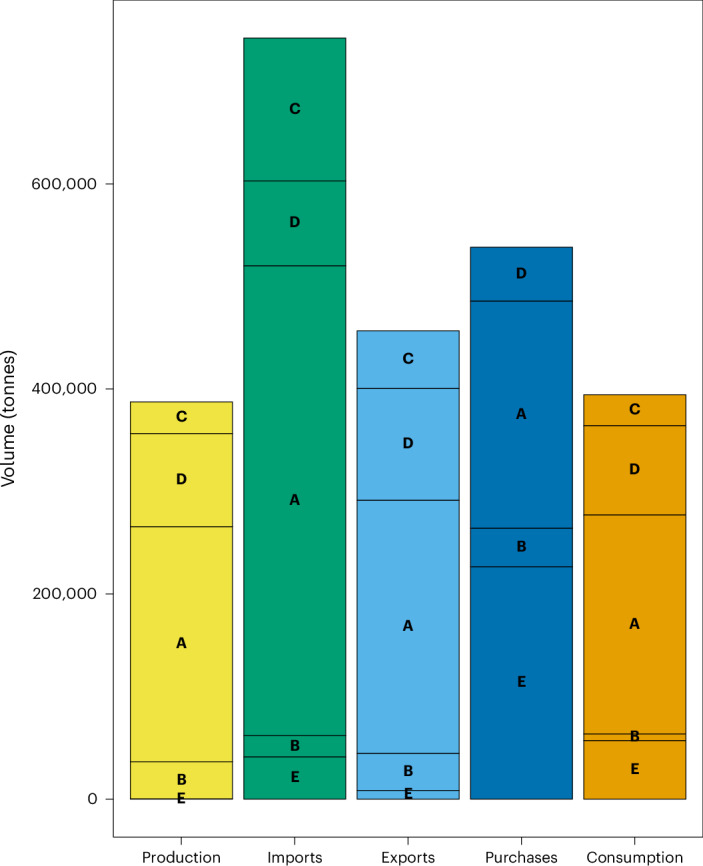
Overview of the long-term annual average of UK seafood production, imports, exports, purchases and consumption. A schematic is shown of the long-term annual average volume (in tonnes) by species type (marine fish (A), molluscs (B), crustaceans (C), diadromous fish (D) and other (E)) between 2009 and 2020. ‘Other’ includes the Food and Agricultural Organization categories freshwater fish and miscellaneous aquatic animals^40^.

Overall, the UK is a net importer of seafood (Fig. 1). As per the International Standard Statistical Classification of Aquatic Animals and Plants, marine fish dominate imports, followed by crustaceans such as prawns—one of the species that we commonly refer to as the ‘big 5’ dominating the UK market (e.g. cod, haddock, salmon, tuna and prawns). Of the species we eat in the UK, 80% is made up of these 5 species^11^. Household food purchase data, which include eating out, revealed that the majority of seafood purchased are fish dishes, such as fish curries and fish pies. Consumption data suggest that most consumed seafood categories are marine fish and molluscs.

**Fig. 2:**
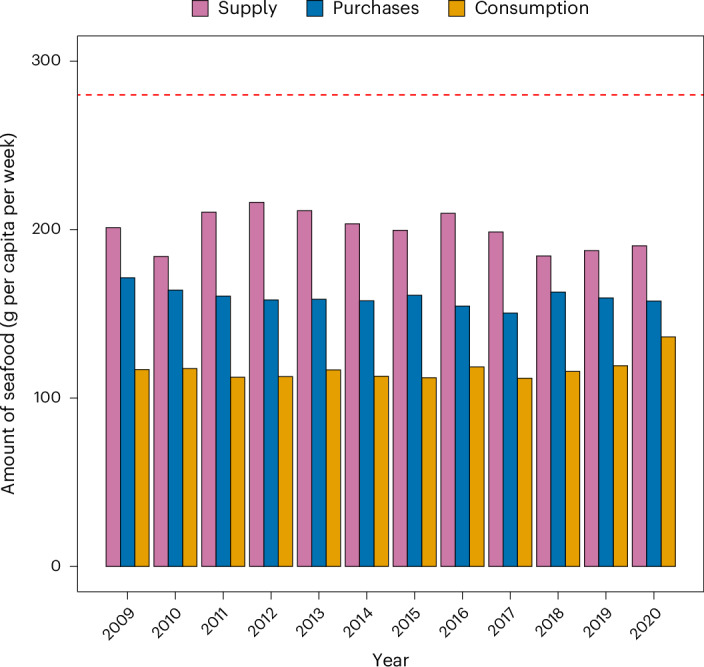
UK annual seafood supplies, purchases and consumption between 2009 and 2020. Supplies refer to production plus imports minus exports. The red dashed line denotes the UK dietary recommendation for finfish consumption for adults (that is, 2 portions a week, 1 of which should be oily fish, with a portion being 140 g (ref. ^14^)).

We found that total seafood supplies (production plus imports less exports), purchases and consumption were relatively stable between 2009 and 2020 (Fig. 2). Our calculations revealed that dietary recommendations for fish consumption in the UK, which are for finfish (that is, lean and oily fish) only and exclude shellfish and molluscs, crustaceans and miscellaneous aquatic animals^14^, are not achieved—in fact, consumption is less than half of what is advised. Total seafood supplies (that is, finfish, shellfish and ‘other’ fish combined) also do not satisfy the dietary recommendation for fish consumption. Estimated UK fish consumption was higher in 2020, at around 136 g per capita per week of seafood, compared with an average of around 115 g per capita per week of seafood in other years. This may be explained by the use of a different methodology to assess dietary intake and the fact that in 2020 there was a higher proportion of women and a lower proportion of children who completed the dietary recalls. On average, and across all years, 145 g per capita per week of seafood was purchased at the household level, compared with 14 g per capita per week of seafood purchased outside the household.

**Fig. 3:**
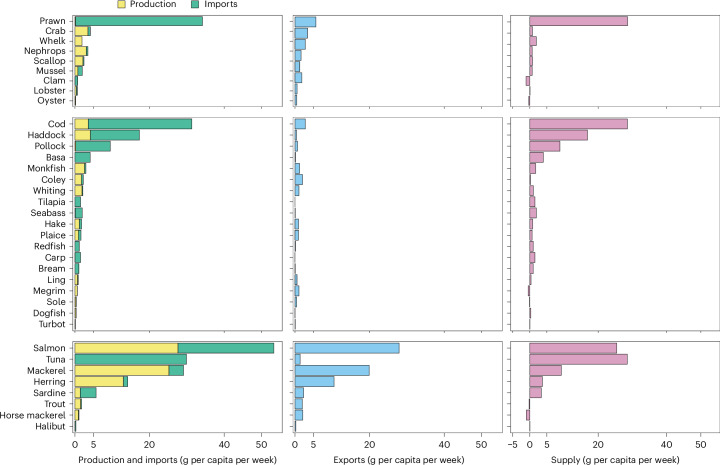
UK seafood production, imports, exports and supplies by species in 2020. Supplies refers to production plus imports minus exports. The list includes selected species for which there are corresponding production, import, and export data, and also includes additional commercially important species that are in the top 30 of supplied species (36 species of a total of 73 species in the database).

UK seafood supplies, production, imports and exports varied considerably between species in 2020 (Fig. 3). Seafood supplies were largest for prawn, cod, salmon and tuna, with more than 20 g per capita per week available to consumers, followed by haddock, pollock and mackerel, with more than 5 g per capita per week available to consumers. Production values were highest for oily fish, in particular for species that the UK exports the most, including salmon, mackerel and herring; approximately 20 g per capita per week of these species was produced in 2020. Total seafood production has been relatively consistent between 2009 and 2020, reaching a peak of 130 g per capita per week in 2014, although population growth increased by 6% during this period. Domestic production of finfish, and of shellfish and other fish, did not satisfy dietary recommendations, nor did it satisfy demand (for example, purchase of finfish, shellfish and other fish combined), indicating that the UK is not self-sufficient for fish (Supplementary Fig. 1). Prawns, cod, haddock, tuna and salmon made up the majority of imported fish: 35.3 g per capita per week, 29.4 g per capita per week, 14.1 g per capita per week, 29.9 g per capita per week and 24.8 g per capita per week, respectively. Unsurprisingly, the most imported fish are also the big five. Seafood exports were dominated by oily fish, in particular, salmon (28.7 g per capita per week), mackerel (19.1 g per capita per week) and herring (14.5 g per capita per week).

### Nutrient balance calculations based on trade

**Fig. 4:**
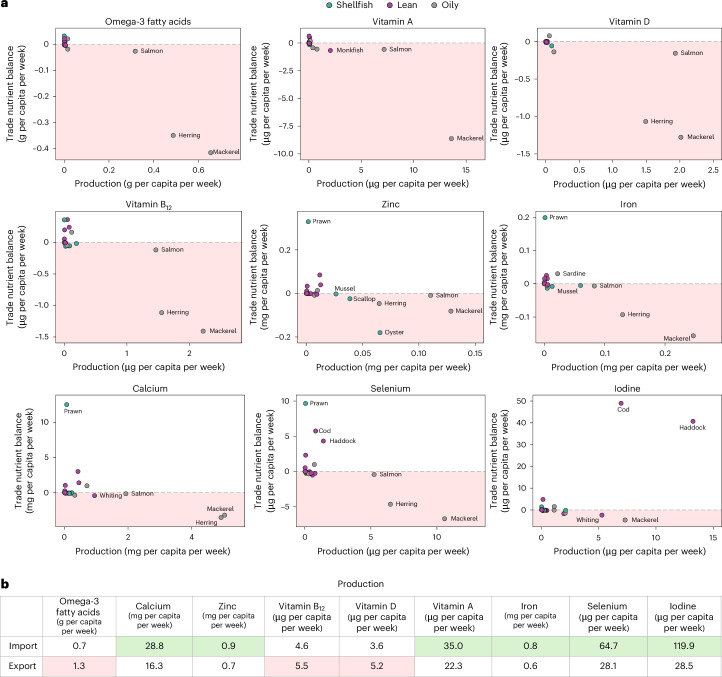
Gains and losses of seafood-relevant nutrients through imports and exports. **a**, The gains and losses in seafood-relevant nutrients (omega-3 fatty acids, calcium, zinc, vitamin B_12_, vitamin D, vitamin A, iron, selenium and iodine) arising through trade versus production across seafoods and groups (for example, lean fish, oily fish and shellfish) in 2020. Trade nutrient balance is calculated as imports minus exports. The shaded red area below zero highlights a negative trade nutrient balance, indicating nutrient losses from the UK. Only species with corresponding production, import, export and nutrient data are included (this equates to 22 species of a total of 73 species in the database). **b**, Summed imports and exports in 2020 for each nutrient. The red shaded areas indicate summed nutrient exports > imports; the green shaded areas indicate summed nutrient imports > exports. Calculations were based on species with corresponding import, export and nutrient data (this varied between nutrients, for example, omega-3 fatty acids (*n* = 26), calcium (*n* = 26), zinc (*n* = 26), vitamin B_12_ (*n* = 26), vitamin D (*n* = 18), vitamin A (*n* = 21), iron (*n* = 26), selenium (*n* = 26) and iodine (*n* = 26)).

Trade (imports minus exports) resulted in a negative nutrient balance in three of the nine seafood-relevant nutrients: greater volumes of omega-3 fatty acids, vitamin B_12_ and vitamin D were exported out of the UK than imported (Fig. 4). Nearly twice as much omega-3 fatty acids were exported than were imported. Such nutrient losses were primarily through exports of oily fish, namely, salmon, mackerel and herring. Notable net gains of calcium, selenium and iodine were detected, attributable to imports of prawns and lean fish, such as cod and haddock.

### Contributions of the seafood-relevant nutrients to reference nutrient intakes

**Fig. 5:**
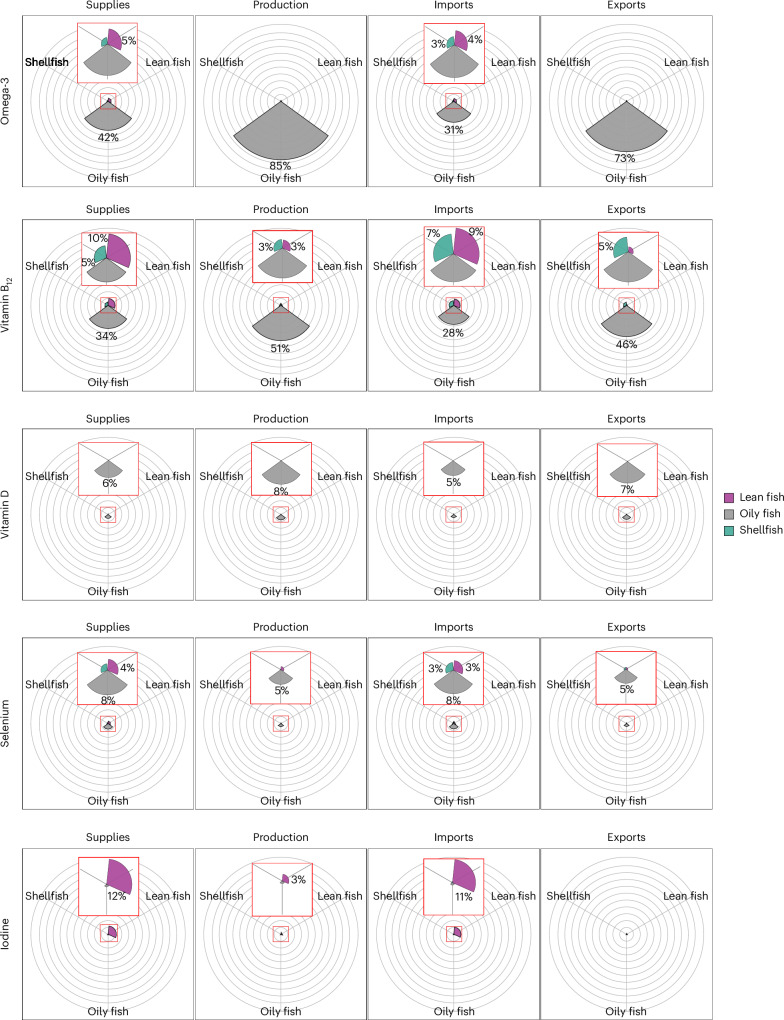
Percentage contributions of seafood-relevant nutrients supplied, produced, imported and exported to weekly RNIs in 2020. The contributions to weekly RNIs by oily fish, lean fish and shellfish are shown. The included nutrients (omega-3 fatty acids, vitamin B_12_, vitamin D, selenium and iodine) are those for which contributions towards RNIs are >2% for either oily fish, lean fish or shellfish supplied, produced, imported and exported. The red boxes indicate enlarged views of the chart. The European recommendation for omega-3 fatty acids is 1,750 mg per week (ref. ^49^). The RNI for vitamin B_12_ is 10.5 µg per week; for vitamin D, 70 µg per week; for selenium, 490 µg per week; and for iodine, 980 µg per week (ref. ^14^). Nutrients present in other fish contributed minimally towards the RNI for the UK adult population.

Contributions of seafood-relevant nutrients (omega-3 fatty acids, vitamin B_12_, vitamin D, selenium and iodine) from supplied, produced, imported and exported fish towards weekly reference nutrient intakes (RNIs) varied between species group but were highest for oily fish (Fig. 5). Of all species produced, only oily fish contributed substantially towards the RNI of omega-3 fatty acids and vitamin B_12_—oily fish supplies alone contributed to nearly half the European recommendation for omega-3 fatty acids and a third towards the RNI of vitamin B_12_. Production contributed considerably to supplies, but most was then exported. Lean fish supplies, mostly from imports, contributed around 10% of the RNI for vitamin B_12_ and iodine. Despite high imports of shellfish, particularly prawns, supplies contributed 5% or less towards the RNI across all nutrients.

## Discussion

We provide a high-resolution perspective on UK seafood supplies and nutrient flows at the species level, incorporating data on production, trade and consumption. This study complements low-resolution global perspectives on seafood trade and relevant seafood nutrients^8^ and asks how the UK, as a case study for high-income countries worldwide that drive international trade patterns and that have a high dependency on seafood imports, could restructure its high-income seafood systems to aid a transition to more sustainable and healthy diets that include seafood. Through mapping UK seafood volumes and nutrient supplies, we showed that current UK seafood supplies do not satisfy dietary recommendations and current UK production does not satisfy dietary recommendations or demand. We confirm that the UK is a net importer of seafood, with prawns, cod, haddock, tuna and salmon—all the fish included in the big five of the most consumed fish in the UK—making up the majority of imported fish. We also found that through imports of prawns, cod and haddock, the UK experiences net gains in selenium and iodine, although the contribution towards the RNI of these nutrients is relatively small. We also show that the UK undergoes large losses of omega-3 fatty acids, vitamin B_12_ and vitamin D through international trade, most notably through exports of oily fish such as salmon, herring and mackerel. Retaining more exports of oily fish for domestic consumption could contribute towards achieving the European recommendation for omega-3 fatty acids (up to 73%) and RNIs for vitamin B_12_ (up to 46%) and vitamin D (up to 7%), which would be beneficial for population health. Increased omega-3 fatty acid levels, which reflect oily fish consumption, are associated with a significantly lower risk of premature death from cardiovascular disease, cancer and other causes^15^. Vitamin B_12_ deficiency is relatively common, with important clinical consequences. The RNI for vitamin B_12_ is difficult to achieve when dietary intake of vitamin-B_12_-containing animal-derived foods is restricted^16^. Therefore, both oily fish and shellfish could fill an important dietary gap as meat consumption may decrease as part of more healthy and sustainable dietary scenarios^3^.

Increased seafood consumption could potentially alleviate emerging micronutrient deficiencies for specific population groups as we transition to net zero diets^17^. In the UK, low intakes of calcium, iron, vitamin A, vitamin D, zinc and iodine are of concern in some children and adolescents; there is also evidence of poor status for vitamin D, and to a lesser extent for iron and vitamin B_12_, in younger women and in those aged 75 years and over^18^. Foods that are important sources of these nutrients are mostly of animal origin. Consumption of plant-based diets, low in animal products, is increasingly recommended^3^; however, such diets can be insufficient in omega-3 fatty acids, vitamin B_12_, vitamin D, calcium, iron and zinc^17^. Seafood consumption could offer the nutrients that plant-based diets lack while minimizing environmental cost^19^, with oily fish species being the most nutritious and having the lowest emissions of all seafood globally^5,20,21^. The UK is a large producer of herring and mackerel, which are relatively cheap for consumers to purchase. These species could substantially contribute to the provision of omega-3 fatty acids, which form the basis of the recommendation to consume one portion of oily fish per person per week^14^. However, most herring and mackerel are exported (Fig. 4), indicating that consumer preferences and demand, rather than a limited supply, determine current patterns of fish production, trade and consumption. This includes consumption levels that fall below recommendations^13^. Indeed, the potential for low emission seafoods to contribute to healthy and sustainable diets depends on consumer preference for those products. UK consumers have a strong preference for a narrow range of species, which are often not predominantly caught or farmed in the UK, including salmon, cod, haddock, tuna and prawns^11^. This is a main driver for the high import levels of these species into the UK^13^. Increased consumption of species such as mackerel and herring would improve the intake of nutrients specifically present in oily fish, such as omega-3 fatty acids, vitamin B_12_ and vitamin D (Fig. 5). Although there is currently little appetite for locally caught small pelagic species, this has not always been the case^22^. Innovation in the production of healthy fish products, such as bivalve products^23^, could encourage dietary shifts; however, such developments need to be cost-sensitive—cost, taste and food safety are still the three most important factors that drive European consumers’ food purchases^24^. Aligning health and environmental advice in relation to fish consumption is also required to avoid confusion for consumers^25^. In addition, policies promoting consumption of locally caught, underused species through improved marketing and product development are also needed^11,21^. Furthermore, the sustainability of the fish stocks needs to be considered. For example, disputes over the management of the Northeast Atlantic mackerel stock has had a negative impact on its sustainability, resulting in Northeast Atlantic mackerel losing its Marine Stewardship Council certification in 2019^26^.

The beneficial effects of fish consumption on health have historically been attributed to its content of omega-3 fatty acids, although fish also plays an important role in the provision of dietary needs for protein, vitamins and minerals^27^. With the European recommendation for omega-3 fatty acids achieved by as little as 152 g (just over 1 portion) of salmon, 47 g (less than a third of a portion) of herring and 67 g (less than half a portion) of mackerel, we could challenge the current UK seafood dietary recommendations. We could argue for more targeted recommendations that focus on locally caught oily fish species to optimize nutrient intake while reducing current recommended portion sizes to better align with current intakes. For example, prawns are one of the most consumed species in the UK, contributing the most towards calcium, iron, selenium and zinc nutrient balances. Yet, shellfish are not included in UK dietary recommendations. Reported seafood consumption in the UK is low and has been low over the past two decades, with intakes less than half of what is advised^27^. In the Netherlands, the dietary recommendations for fish consumption were revised to make them seem more achievable^28^. Moreover, dietary guidelines should consider sustainability of seafood supplies^29^ and encourage a greater diversity of seafood species to be consumed^30^. Diversifying the intake of seafood consumed may make the seafood supply chain more resilient by reducing potential variability in production from external shocks, such as global pandemics, extreme weather events and supply chain disruptions^31^. The UK relies heavily on imports for consumption, leaving it open to risks such as changes in trade agreements and changes in tariffs^11^. However, a combination of low consumer demand, high diversity of supply chains and high purchasing power suggests that the UK would be able to cope with most shocks to the aquatic food supply chains in terms of food and nutrient security^13^. Perhaps it is time that dietary recommendations go beyond health implications and be more context specific, such as providing guidelines for specific population groups depending on where most gains could be made^5^. From that perspective, individuals reporting low or no seafood intake will benefit the most from seafood consumption^4^.

For high-income countries that have a high dependency on imports to satisfy consumer demand for seafood, international trade patterns are driven by economic value rather than nutrient supply. For example, an estimated 40% of farmed fish is currently exported, with salmon being the largest food export from Scotland and the UK more widely, whereas most salmon for human consumption is imported^32^. In the UK, the amount of seafood imported varies considerably by year. In 2022, the UK imported most fish from Norway, Iceland and China (mostly cod, haddock and salmon), but also imported large amounts of tuna, mainly from Ecuador, and prawns from Vietnam and India^33^. In the UK, imports of oily fish, such as salmon, contributed to the nutrient balance of omega-3 fatty acids (31% of RNI), vitamin B_12_ (28% of RNI), selenium (8% of RNI), and vitamin D (5% of RNI), and imports of shellfish, such as prawns, contributed considerably to the nutrient balance of vitamin B_12_ (7%) and, to a lesser extent, other micronutrients (Fig. 5). Exporting countries such as Vietnam and India may benefit economically from trade in prawns with the UK. But both countries have a high burden of malnutrition and exports of important nutrients could therefore disproportionally affect their nutrient security, especially in Vietnam where the contribution of fish and seafood to dietary energy and protein intake is high^8^. It has been proposed that decision makers should consider nutrients from fisheries and aquaculture a key resource for tackling malnutrition, one that needs protection in trade policies and fishing agreements^8^. Indeed, the potential of fisheries to tackle micronutrient deficiencies has been widely acknowledged^34,35^, especially in low-income countries where such deficiencies are more prevalent^36^. For instance, small pelagic fish offer affordable micronutrients to low- and middle-income countries^37^ and, recently, the Blue Food Assessment found that facilitating increased consumption of aquatic foods in vulnerable African and South American populations could address vitamin B_12_ and omega-3 deficiencies^38^. For the UK, and possibly other high-income countries that drive international trade patterns, higher consumption of domestically produced fish species will not only improve national nutrient trade balances but also reduce pressures on aquatic stocks that are vital for low- and middle-income countries from a nutritional perspective.

We acknowledge some limitations to our database. First, aggregating data from different sources provides its own challenges; for example, the Marine Management Organisation (MMO) and HM Revenue and Customs (HMRC) define foreign fleets differently. This is probably due to inaccurate trade data, as not all seafood landed by UK fleets outside of the UK go through customs and thus are not captured by the HMRC, and differences in the conversion factors used by the HMRC and MMO for the same species. Second, exact volumes of seafood in cold storage and exact volumes wasted at different levels in the supply chain are unknown. Large amounts of pelagic fish, notably herring and mackerel, are held in cold storage throughout the year due to the seasonal nature of these fisheries. Also, although nutrient composition was selected for raw species to allow for comparisons across the dataset, we acknowledge that cooking may affect nutrient composition in seafood^39^. Moreover, granularity differs between databases, especially concerning fish groups, species and subspecies. This makes it difficult to ascertain the full picture of nutrient flows because not all species had corresponding data.

## Conclusion

There is currently a mismatch between what is being produced and consumed in the UK. Consequently, the UK experiences large nutrient losses from international seafood exports. We highlight potential opportunities to reshape our seafood supply chains by proposing increased consumption of locally caught species, such as herring and mackerel, that optimize nutrient intake, while considering ways to reduce climate emissions and reduce pressures on aquatic stocks from nutritionally vulnerable nations. As seafood is a global commodity, the extensive mapping of UK seafood supplies will reveal examples for optimizing seafood supply chains in other high-income countries that are also highly reliant on trade. Understanding how national seafood consumption relates to food supply chains and global trade is essential to help develop both fisheries and public health policies that promote the consumption of locally produced species. Furthermore, developing innovative, healthy seafood products may nudge consumers towards increasing and diversifying fish intake as we transition to net zero.

## Methods

We created a unique database linking seafood production (capture and aquaculture), trade (imports and exports), purchases (both within and out of home) and UK consumption data to the species level, with data obtained between 2009 and 2020 (the most recent year for which all datasets were available). The database includes species caught in marine, fresh and brackish water. Species were allocated a species categories as per the Food and Agricultural Organization’s International Standard Statistical Classification for Aquatic Animals and Plants^40^ and were classified as oily, lean or shellfish as per the Scientific Advisory Committee in Nutrition (Supplementary Table 1)^41^ to facilitate comparisons with the UK dietary guidelines and fish consumption.

### Data sources

#### UK capture and aquaculture production

MMO landings data were used to calculate the total volumes (in 1,000 tonnes of live weight) of each species landed into the UK and abroad by UK vessels^42^. Annual landings data are provided in the UK sea fisheries statistics reports. All seafoods landed were assumed to be intended for direct human consumption. To calculate the edible portion of the fish landed, appropriate conversion factors were applied^43^. When a conversion factor was not provided, the average conversion factor of the overarching species type (for example, freshwater fish, small pelagic fish, crustaceans and so on) was calculated. Total volumes (tonnes of live weight) of farmed seafood produced in UK waters were obtained from the Centre for Environment, Fisheries and Aquaculture Science (personal communications). Conversion factors were applied to estimate the weight of the edible portion. Cleaner fish and fish intended for coarse fishing were removed from the dataset because they are not intended for human consumption.

#### UK imports and exports of seafood

Annual import and export volume (kg converted to g) of all seafood and products were obtained from HMRC trade data^44^. Trade data were extracted from Harmonised System (HS) commodity codes within sections 03 (fish crustaceans, molluscs and other aquatic invertebrates) and 16 (preparations of meat fish or crustaceans, mollusc or other aquatic invertebrates). Further granularity can be found in Supplementary Table 2.

The commodity for ‘fats and oils and their fractions of fish or marine mammals’ (1503) was removed because the product could not be confirmed to be intended for human consumption. Traded products that fell below a statistical threshold (dictated by weight or monetary value) were aggregated. As a result, large quantities of seafood were assigned to ‘03 HS2 Below Threshold Trade’, for which no species composition data or information on product processing is available. Any over- or underestimation of UK seafood supplies may be attributed to the imported and exported products assigned to this commodity code.

Traded commodities may be whole or may already be prepared in some way. Using the European Market Observatory for Fisheries commodity table^45^, conversion factors were applied to calculate the whole weight. Conversion factors were applied to estimate the weight of the edible portion^43^. Landings of fish into the UK by foreign vessels are typically included in import statistics. Fish landed into the UK by foreign vessels that have a final destination outside the UK are not included in the import statistics. Similarly, landings of fish by UK vessels abroad count as exports. Trans-shipments refer to fish that are landed in the UK but are transported (by road or ferry) out of the UK before it is sold. This may occur when the owner takes advantage of higher market prices for some fish species when sold at continental markets rather than in the UK.

#### UK seafood purchases

Average weekly per capita UK seafood purchases (g) were obtained from the Department for Environment, Food and Rural Affairs family food data^46^, including food purchased for UK households and food consumed out of home. This dataset was the least granular, aggregating species under categories such as ‘white fish, fresh or chilled’ and ‘takeaway fish’.

#### UK seafood consumption

UK seafood intake data were obtained from the National Dietary and Nutrition Survey (NDNS) (years 1–12)^18^. Every 2 years, a representative sample of around 500 adults (aged 19+ years) and 500 children (aged 1.5–18 years) complete a 4 day food diary with estimated weights, which details information on seafood species consumed. However, in year 12, NDNS participants collected 4 non-consecutive 24 h dietary recalls using Intake24, an online dietary data collection tool, instead of the paper diary. Also, the 2020 NDNS assessed the impact of the coronavirus disease 2019 pandemic and the sample included individuals who had been involved in previous years. As a result, the NDNS 12 cohort was characterized by a relatively higher proportion of adults and a higher proportion of women completing the dietary recalls. Reported seafood consumption over the 4 days was extracted. Average portion sizes (g) and proportion of respondents consuming seafood were combined with UK population census data from the Office of National Statistics to estimate weekly seafood consumption to the species level where possible. When intakes of products containing seafood (for example, fish pie and sushi) were reported, the portion of fish was estimated.

#### Seafood nutrient composition tables

Seafood nutrient composition data were obtained from Department of Health and Social Care^47^ but complemented with data from Norwegian food composition tables^48^ for species where data from the Department of Health and Social Care were lacking (for the species blue ling, catfish, char, conger eel, cusk, dogfish, eel, garfish, halibut, herring, lemon sole, ling, monkfish, mussel, oyster, perch, pike, powan, redfish, salmon, scallop, shark, skate, sprat, squid, turbot, whiting and winkle). Fish-based nutrients considered most important for human health were selected to be omega-3 fatty acids, vitamin D, vitamin A, vitamin B_12_, calcium, iodine, iron, selenium and zinc. Omega-3 content was estimated by summing eicosapentaenoic and docosahexaenoic fatty acids. Although seafood is not typically consumed raw, nutrient composition was selected for raw species only to allow comparisons across the dataset. Despite combining nutritional composition data from two sources, composition data were not available for all species. Species for which nutritional composition data are lacking are indicated (Supplementary Table 3). RNIs were obtained from Public Health England^14^. Nutrient requirements differ between age groups; thus specific nutrient intake recommendations were obtained for the general adult population. For cases in which separate recommendations were provided based on gender, the average recommendation was calculated. There is currently no RNI for omega-3 fatty acids but the European Food Safety Authority has set an adequate intake of 250 mg for adults per day^49^.

### Calculating UK seafood supplies

UK seafood supplies were calculated as production plus imports less exports. Supplies were calculated for species for which there are corresponding production, import, and export data, and also for additional commercially important species that are in the top 30 of supplied species (36 species of a total of 73 species in the database). For a few species, negative seafood supplies were derived (such as for megrim and horse mackerel). This occurred when the combined production and imports were estimated to be less than exports. It is unlikely that imports were re-exported^42^ but there may be other contributing factors, such as errors mismatching species categories when aggregating capture and aquaculture production and HMRC trade data. Moreover, there is also some disparity between what the HMRC and MMO classify as a ‘foreign’ vessel. Finally, stock variation (that is, seafood to be placed in cold storage at certain points in the supply chain) is common for some fisheries due to their seasonal nature but this was not included when calculating UK seafood supplies.

### Reporting summary

Further information on research design is available in the Nature Portfolio Reporting Summary linked to this article.

## Supplementary information

Supplementary Information Supplementary Fig. 1 and Tables 1–3.

Reporting Summary

## Data Availability

The database created is publicly available on the UK Data Archive: 10.5255/UKDA-SN-856955.

## Code availability

The code is publicly available on GitHub: https://github.com/The-Rowett-Institute/RI-B5-04_Seafood_Database.

## References

[1] Afshin, A. et al. Health effects of dietary risks in 195 countries, 1990–2017: a systematic analysis for the Global Burden of Disease Study 2017. *Lancet* **393**, 1958–1972 (2019). 30954305 10.1016/S0140-6736(19)30041-8 PMC6899507

[2] Crippa, M. et al. Food systems are responsible for a third of global anthropogenic GHG emissions. *Nat. Food* **2**, 198–209 (2021). 37117443 10.1038/s43016-021-00225-9

[3] Willett, W. et al. Food in the Anthropocene: the EAT–*Lancet* Commission on healthy diets from sustainable food systems. *Lancet* **393**, 447–492 (2019). 30660336 10.1016/S0140-6736(18)31788-4

[4] Zheng, J. et al. Fish consumption and CHD mortality: an updated meta-analysis of seventeen cohort studies. *Public Health Nutr.* **15**, 725–737 (2012). 21914258 10.1017/S1368980011002254

[5] Bianchi, M. et al. Assessing seafood nutritional diversity together with climate impacts informs more comprehensive dietary advice. *Commun. Earth Environ.* **3**, 188 (2022).

[6] Horgan, G. W. et al. Achieving dietary recommendations and reducing greenhouse gas emissions: modelling diets to minimise the change from current intakes. *Int. J. Behav. Nutr. Phys. Act.* **13**, 46 (2016). 27056829 10.1186/s12966-016-0370-1 PMC4823893

[7] Watson, R. A. et al. Global seafood trade flows and developing economies: insights from linking trade and production. *Mar. Policy* **82**, 41–49 (2017).

[8] Nash, K. L. et al. Trade and foreign fishing mediate global marine nutrient supply. *Proc. Natl Acad. Sci. USA* **119**, e2120817119 (2022). 35605118 10.1073/pnas.2120817119 PMC9295801

[9] FAO, IFAD, UNICEF, WFP and WHO. *The State of Food Security and Nutrition in the World 2023* (FAO, 2023); 10.4060/cc3017en

[10] FAO. *The State of World Fisheries and Aquaculture 2022* (FAO, 2022); 10.4060/cc0461en

[11] Harrison, L. O. J. et al. Widening mismatch between UK seafood production and consumer demand: a 120-year perspective. *Rev. Fish Biol. Fish.*10.1007/s11160-023-09776-5 (2023). 37360578 10.1007/s11160-023-09776-5 PMC10234684

[12] de Roos, B. Integrated aquaculture–agriculture production supports food and nutrition security in Bangladesh. *Nat. Food* **4**, 833–834 (2023). 37696965 10.1038/s43016-023-00845-3

[13] Jennings, S. et al. Aquatic food security: insights into challenges and solutions from an analysis of interactions between fisheries, aquaculture, food safety, human health, fish and human welfare, economy, and environment. *Fish Fish.* **17**, 893–938 (2016).

[14] *Government Dietary Recommendations. Government Recommendations for Energy and Nutrients for Males and Females Aged 1–18 Years and 19+ Years* (PHE, 2016); https://assets.publishing.service.gov.uk/government/uploads/system/uploads/attachment_data/file/618167/government_dietary_recommendations.pdf

[15] Harris, W. S. et al. Blood n-3 fatty acid levels and total and cause-specific mortality from 17 prospective studies. *Nat. Commun.* **12**, 2329 (2021). 33888689 10.1038/s41467-021-22370-2 PMC8062567

[16] Hunt, A. et al. Vitamin B_12_ deficiency. *BMJ* **349**, g5226 (2014). 25189324 10.1136/bmj.g5226

[17] Beal, T. et al. Estimated micronutrient shortfalls of the EAT–*Lancet* planetary health diet. *Lancet Planet. Health* **7**, e233–e237 (2023). 36889864 10.1016/S2542-5196(23)00006-2

[18] *National Diet and Nutrition Survey* (OHID, 2021); https://www.gov.uk/government/collections/national-diet-and-nutrition-survey

[19] Springmann, M. Eating a nutritionally adequate diet is possible without wrecking long-term health, the planet, or the pocket. *Lancet Planet. Health* **7**, e544 (2023). 37302408 10.1016/S2542-5196(23)00129-8

[20] Hallström, E. et al. Combined climate and nutritional performance of seafoods. *J. Clean. Prod.* **230**, 402–411 (2019).

[21] Robinson, J. P. W. et al. Navigating sustainability and health trade-offs in global seafood systems. *Environ. Res. Lett.* **17**, 124042 (2022).

[22] Dickey-Collas, M. et al. Lessons learned from stock collapse and recovery of North Sea herring: a review. *ICES J. Mar. Sci.* **67**, 1875–1886 (2010).

[23] Gawel, J. P. F. et al. Barriers and drivers to increasing sustainable bivalve seafood consumption in a mass market economy. *Food Front.* **4**, 1257–1269 (2023).

[24] *Making Our Food Fit for the Future—New Trends and Challenges.* Eurobarometer Survey (European Union, 2020); https://europa.eu/eurobarometer/surveys/detail/2241

[25] Clonan, A. et al. The dilemma of healthy eating and environmental sustainability: the case of fish. *Public Health Nutr.* **15**, 277–284 (2012). 21619717 10.1017/S1368980011000930

[26] Østhagen, A. et al. Collapse of cooperation? The North-Atlantic mackerel dispute and lessons for international cooperation on transboundary fish stocks. *Marit. Stud.* **19**, 155–165 (2020).

[27] de Roos, B. et al. The potential impact of compositional changes in farmed fish on its health-giving properties: is it time to reconsider current dietary recommendations? *Public Health Nutr.* **20**, 2042–2049 (2017). 28535834 10.1017/S1368980017000696 PMC10261345

[28] Kromhout, D. et al. The 2015 Dutch food-based dietary guidelines. *Eur. J. Clin. Nutr.* **70**, 869–878 (2016). 27049034 10.1038/ejcn.2016.52 PMC5399142

[29] Lofstedt, A. et al. Less than half of the European dietary recommendations for fish consumption are satisfied by national seafood supplies. *Eur. J. Nutr.* **60**, 4219–4228 (2021). 33999272 10.1007/s00394-021-02580-6 PMC8572203

[30] Golden, C. D. et al. Aquatic foods to nourish nations. *Nature* **598**, 315–320 (2021). 34526720 10.1038/s41586-021-03917-1 PMC10584661

[31] Lester, S. E. et al. The role of marine aquaculture in contributing to the diversity and stability of U.S. seafood production. *Mar. Policy* **160**, 105994 (2024).

[32] *Food and Drink Supply Chain and Transport Industry: Evidence Report* (Scottish Government, 2019); https://www.gov.scot/publications/identifying-options-developing-transport-infrastructure-food-drink-supply-chain-strengthen-resilience/

[33] *UK Sea Fisheries Annual Statistics Report 2022* (MMO, 2023); https://www.gov.uk/government/statistics/uk-sea-fisheries-annual-statistics-report-2022

[34] Hicks, C. C. et al. Harnessing global fisheries to tackle micronutrient deficiencies. *Nature* **574**, 95–98 (2019). 31554969 10.1038/s41586-019-1592-6

[35] Golden, C. D. et al. Nutrition: fall in fish catch threatens human health. *Nature* **534**, 317–320 (2016). 27306172 10.1038/534317a

[36] Han, X. et al. Global, regional, and national burdens of common micronutrient deficiencies from 1990 to 2019: a secondary trend analysis based on the Global Burden of Disease 2019 study. *eClinicalMedicine* **44**, 101299 (2022). 35198923 10.1016/j.eclinm.2022.101299 PMC8850322

[37] Robinson, J. P. W. et al. Small pelagic fish supply abundant and affordable micronutrients to low- and middle-income countries. *Nat. Food* **3**, 1075–1084 (2022). 37118295 10.1038/s43016-022-00643-3

[38] Crona, B. I. et al. Four ways blue foods can help achieve food system ambitions across nations. *Nature* **616**, 104–112 (2023). 36813964 10.1038/s41586-023-05737-x PMC10076219

[39] Sobral, M. M. C. et al. Domestic cooking of muscle foods: impact on composition of nutrients and contaminants. *Compr. Rev. Food Sci. Food Saf.* **17**, 309–333 (2018). 33350087 10.1111/1541-4337.12327

[40] *International Standard Statistical Classification of Aquatic Animals and Plants (ISSCAAP)* (FAO, 2000); https://www.fao.org/fishery/docs/DOCUMENT/cwp/handbook/annex/AnnexS2listISSCAAP2000.pdf

[41] SACN. *Advice on Fish Consumption: Benefits and Risks* (The Stationery Office, 2004); https://assets.publishing.service.gov.uk/media/5a7dbedc40f0b65d88634277/SACN_Advice_on_Fish_Consumption.pdf

[42] *UK Sea Fisheries Annual Statistics* (MMO, 2023); https://www.gov.uk/government/collections/uk-sea-fisheries-annual-statistics

[43] Hilborn, R. et al. The environmental cost of animal source foods. *Front. Ecol. Environ.* **16**, 329–335 (2018).

[44] HMRC. UK Trade Info. https://www.uktradeinfo.com/search/traders/ (2023).

[45] *European Market Observatory for Fisheries and Aquaculture Products.* Metadata 2—Data management (EUMOFA, 2023); https://eumofa.eu/metadata

[46] *Family Food Statistics* (DEFRA, 2023); https://www.gov.uk/government/collections/family-food-statistics

[47] *Nutrient Analysis of Fish and Fish Products* (DoH, 2013); https://www.gov.uk/government/publications/nutrient-analysis-of-fish

[48] Norwegian Food Composition Database. *Norwegian Food Safety Authority* http://www.matvaretabellen.no (2012).

[49] EFSA Panel on Dietetic Products, Nutrition and Allergies (NDA).Scientific opinion on dietary reference values for fats, including saturated fatty acids, polyunsaturated fatty acids, monounsaturated fatty acids, *trans*fatty acids, and cholesterol. *EFSA J.* **8**, 1461 (2010).

