# Supplementary material for: Seafood supply mapping reveals production and consumption mismatches and large dietary nutrient losses through exports in the United Kingdom
Source: Nat Food. 2025 Jan 2;6(3):244–52. doi: 10.1038/s43016-024-01102-x (PMC11932926; doi:10.1038/s43016-024-01102-x)
Supplement: Supplementary file 1 — Supplementary Fig. 1 and Tables 1–3. [file 43016_2024_1102_MOESM1_ESM.pdf]

nature food

Supplementary information

https://doi.org/10.1038/s43016-024-01102-x

Seafood supply mapping reveals production and consumption mismatches and large dietary nutrient losses through exports in the United Kingdom

In the format provided by the authors and unedited

Supplementary Information

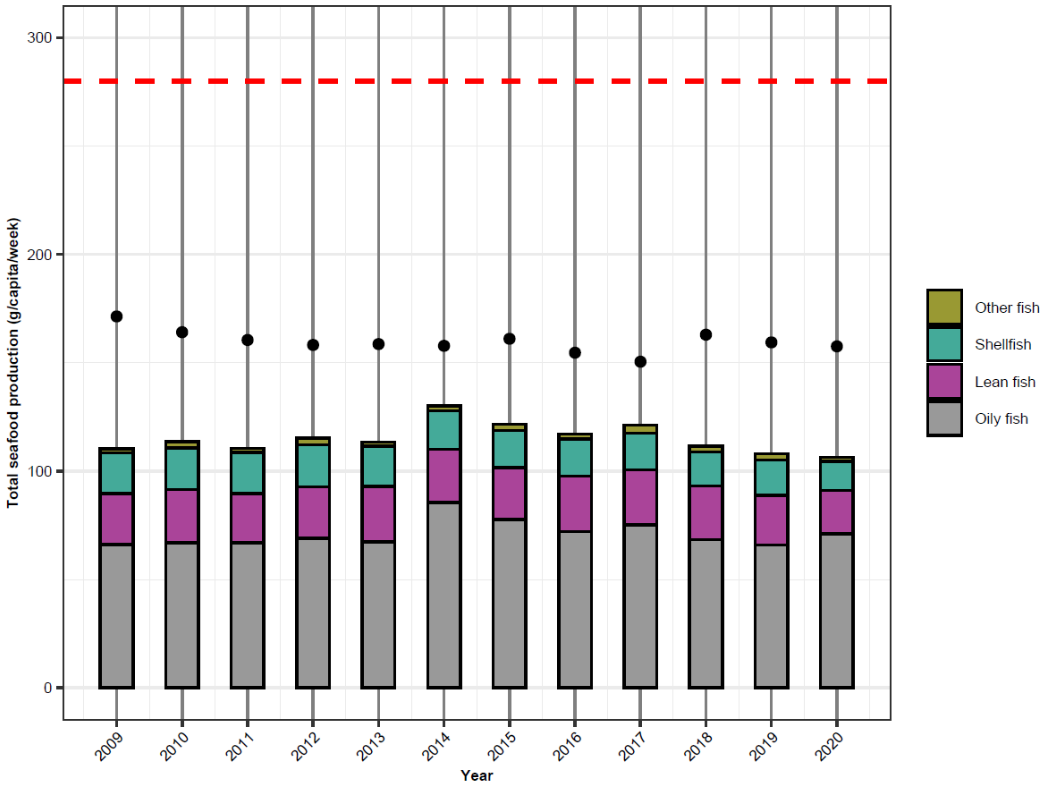

SI Figure 1. UK annual domestic seafood production (g/capita/week) for oily fish, lean fish, shellfish, and "other fish", between 2009 and 2020. The red dotted line denotes the UK dietary recommendation for finfish consumption in adults (e.g., 280 g/capita/week). The black circles denote seafood purchases (g/capita/week) of oily fish, lean fish, shellfish, and "other fish" combined, between 2009 and 2020.

SI Table 1. Species classification as per the Scientific Advisory Committee in Nutrition (SACN).

Lean fish	Barbel, Basa, Blue whiting, Bream, Brill, Carp, Chub, Cobia, Cod, Coley, Cusk, Cusk-eel, Dab, Dogfish, Fish dish (of lean origin), Flounder, Grenadier, Gurnard, Haddock, Hake, John dory, Lemon sole, Ling, Megrim, Monkfish, Nile perch, Orfe, other freshwater fish (of lean origin), other marine fish (of lean origin), Perch, Pike, Pike-perch, Plaice, Pollock, Pomfret, Pouting, Ray, Red mullet, Redfish, Roach, Rudd, Scabbardfish, Seabass, Skate, Sole, Tench, Tilapia, Toothfish, Turbot, Weever, Whiting, Witch
Oily fish	Anchovy, Char, Eel, Fish dish (of oily origin), Halibut, Herring, Horse mackerel, Mackerel, other marine fish (of oily origin), Picarel, Pilchard, Salmon, Sandeel, Sardine, Smelt, Sprat, Swordfish, Trout, Tuna
Shellfish	Abalone, Clam, Cockle, Crab, Crayfish, Lobster, Mussel, Nephrops, other molluscs, Oyster, Prawn, Scallop, Whelk
Other fish	Cuttlefish, Jellyfish, Octopus, other molluscs and aquatic invertebrates, other fish products, Sea cucumber, Sea urchin, Squid

SI Table 2. Harmonised System (HS) commodity codes within sections 03 (Fish crustaceans, molluscs, and other aquatic invertebrates) and 16 (Preparations of meat fish or crustaceans, molluscs, or other aquatic invertebrates) for extracting trade data.

03 HS2	HS2 Below Threshold Trade
0302	Fish, fresh or chilled (excl. fish fillets and other fish meat of heading 0304)
0303	Frozen fish (excl. fish fillets and other fish meat of heading 0304)
0304	Fish fillets and other fish meat, whether or not minced, fresh, chilled or frozen
0305	Fish, fit for human consumption, dried, salted or in brine; smoked fish, fit for human consumption, whether or not cooked before or during the smoking process; flours, meals and pellets of fish, fit for human consumption
0306	Crustaceans, whether in shell or not, live, fresh, chilled, frozen, dried, salted or in brine, even smoked, incl. crustaceans in shell cooked by steaming or by boiling in water; flours, meals, and pellets of crustaceans, fit for human consumption
0307	Molluscs, fit for human consumption, even smoked, whether in shell or not, live, fresh, chilled, frozen, dried, salted or in brine; flours, meals, and pellets of molluscs, fit for human consumption
0308	Aquatic invertebrates other than crustaceans and molluscs, live, fresh, chilled, frozen, dried, salted or in brine, even smoked; flours, meals, and pellets of aquatic invertebrates other than crustaceans and molluscs, fit for human consumption
1604	Prepared or preserved fish; caviar and caviar substitutes prepared from fish eggs
1605	Crustaceans, molluscs, and other aquatic invertebrates prepared or preserved (excl. smoked)

SI Table 3. Species for which nutritional composition data are lacking.

Lean fish	Barbel, Basa, Blue whiting, Bream, Brill, Carp, Chub, Cobia, Cusk, Cusk-eel, Dab, Fish dish (of lean origin), Flounder, Grenadier, Gurnard, Hake, John dory, Megrim, Nile perch, Orfe, other freshwater fish (of lean origin), other marine fish (of lean origin), Perch, Pike, Pike-perch, Pomfret, Pouting, Ray, Red mullet, Roach, Rudd, Scabbardfish, Skate, Tench, Tilapia, Toothfish, Weever, Witch
Oily fish	Anchovy, Fish dish (of oily origin), Horse mackerel, other marine fish (of oily origin), Picarel, Pilchard, Sandeel, Smelt, Swordfish
Shellfish	Abalone, Clam, Cockle, Crayfish, Lobster, Nephrops, other molluscs, Whelk
Other fish	Cuttlefish, Jellyfish, Octopus, other molluscs and aquatic invertebrates, other fish products, Sea cucumber, Sea urchin
