# Supplementary material for: Seafood supply mapping reveals production and consumption mismatches and large dietary nutrient losses through exports in the United Kingdom
Source: Nat Food. 2025 Jan 2;6(3):244–52. doi: 10.1038/s43016-024-01102-x (PMC11932926; doi:10.1038/s43016-024-01102-x)
Supplement: Supplementary file 2 — Reporting Summary [file 43016_2024_1102_MOESM2_ESM.pdf]

nature portfolio

Corresponding author(s): *Baukje de Roos*

Last updated by author(s): *Nov 11, 2024*

Reporting Summary

Nature Portfolio wishes to improve the reproducibility of the work that we publish. This form provides structure for consistency and transparency in reporting. For further information on Nature Portfolio policies, see our Editorial Policies and the Editorial Policy Checklist.

Statistics

For all statistical analyses, confirm that the following items are present in the figure legend, table legend, main text, or Methods section.

n/a	Confirmed	
☐	☒	The exact sample size (n) for each experimental group/condition, given as a discrete number and unit of measurement
☒	☐	A statement on whether measurements were taken from distinct samples or whether the same sample was measured repeatedly
☒	☐	The statistical test(s) used AND whether they are one- or two-sided<br>*Only common tests should be described solely by name; describe more complex techniques in the Methods section.*
☒	☐	A description of all covariates tested
☒	☐	A description of any assumptions or corrections, such as tests of normality and adjustment for multiple comparisons
☒	☐	A full description of the statistical parameters including central tendency (e.g. means) or other basic estimates (e.g. regression coefficient) AND variation (e.g. standard deviation) or associated estimates of uncertainty (e.g. confidence intervals)
☒	☐	For null hypothesis testing, the test statistic (e.g. F, t, r) with confidence intervals, effect sizes, degrees of freedom and P value noted<br>*Give P values as exact values whenever suitable.*
☒	☐	For Bayesian analysis, information on the choice of priors and Markov chain Monte Carlo settings
☒	☐	For hierarchical and complex designs, identification of the appropriate level for tests and full reporting of outcomes
☒	☐	Estimates of effect sizes (e.g. Cohen's d, Pearson's r), indicating how they were calculated

Our web collection on statistics for biologists contains articles on many of the points above.

Software and code

Policy information about availability of computer code

Data collection	*We created a unique database linking seafood production (capture and aquaculture), trade (imports and exports), purchases (both within and out-of-home), and UK consumption data, to species level, with data obtained between 2009 and 2020 (the most recent year all datasets were available to).*
Data analysis	*Data analysis was completed in R (Version 4.3.2). UK seafood supplies were calculated as production plus imports, less exports. The database created is publicly available on the UK Data Archive: https://doi.org/10.5255/UKDA-SN-856955. The code is publicly available on GitHub: https://github.com/The-Rowett-Institute/RI-B5-04_Seafood_Database*

For manuscripts utilizing custom algorithms or software that are central to the research but not yet described in published literature, software must be made available to editors and reviewers. We strongly encourage code deposition in a community repository (e.g. GitHub). See the Nature Portfolio guidelines for submitting code & software for further information.

Data

Policy information about availability of data

All manuscripts must include a data availability statement. This statement should provide the following information, where applicable:

- Accession codes, unique identifiers, or web links for publicly available datasets
- A description of any restrictions on data availability
- For clinical datasets or third party data, please ensure that the statement adheres to our policy

Marine Management Organization (MMO) landings data were used to calculate the total volumes (thousand tonnes [live weight]) of each species landed into the UK and abroad by UK vessels. Total volumes (tonnes [live weight]) of farmed seafood produced in UK waters 387 were obtained from the Centre for Environment, Fisheries and Aquaculture Science (CEFAS). Annual import and export volume (kg converted to g) of all seafood and products were obtained from HM Revenue and Customs (HMRC) trade data. Conversion factors were applied to estimate the amount of seafood intended for human consumption. Average weekly per capita UK seafood purchases (g) were obtained from the Department for Environment, Food and Rural Affairs (DEFRA) family food data. UK seafood intake data were obtained from the National Dietary and Nutrition Survey (NDNS) (years 1 to 12). Seafood nutrient composition data were obtained from Department of Health and Social Care (DHSC). but complemented with data from Norwegian food composition tables for species where data from DHSC were lacking. The database created will be made publicly available on the UK Data Archive (https://doi.org/10.5255/UKDA-SN-856955).

Research involving human participants, their data, or biological material

Policy information about studies with human participants or human data. See also policy information about sex, gender (identity/presentation), and sexual orientation and race, ethnicity and racism.

Reporting on sex and gender	*N/A*
Reporting on race, ethnicity, or other socially relevant groupings	*N/A*
Population characteristics	*N/A*
Recruitment	*N/A*
Ethics oversight	*N/A*

Note that full information on the approval of the study protocol must also be provided in the manuscript.

Field-specific reporting

Please select the one below that is the best fit for your research. If you are not sure, read the appropriate sections before making your selection.

☐ Life sciences ☐ Behavioural & social sciences ☒ Ecological, evolutionary & environmental sciences

For a reference copy of the document with all sections, see nature.com/documents/nr-reporting-summary-flat.pdf

Life sciences study design

All studies must disclose on these points even when the disclosure is negative.

Sample size	*N/A*
Data exclusions	*N/A*
Replication	*N/A*
Randomization	*N/A*
Blinding	*N/A*

Behavioural & social sciences study design

All studies must disclose on these points even when the disclosure is negative.

Study description	*N/A*
Research sample	*N/A*

Sampling strategy	*N/A*
Data collection	*N/A*
Timing	*N/A*
Data exclusions	*N/A*
Non-participation	*N/A*
Randomization	*N/A*

Ecological, evolutionary & environmental sciences study design

All studies must disclose on these points even when the disclosure is negative.

Study description	*We aimed to obtain an integrated and cross-sectoral understanding of how seafood consumption in the UK relates to food supply chains - what is being produced, what is imported and exported, and how this relates to what we eat and what we should be eating for optimal health. For this, we created a unique database mapping seafood production (capture and aquaculture), trade (imports and exports), purchases (within and out-of-home), and seafood consumption, to species level, between 2009 and 2020.*
Research sample	*Marine Management Organisation (MMO) landings data were used to calculate the total volumes (thousand tonnes [live weight]) of each species landed into the UK and abroad by UK vessels. Total volumes (tonnes [live weight]) of farmed seafood produced in UK waters 387 were obtained from the Centre for Environment, Fisheries and Aquaculture Science (CEFAS). Annual import and export volume (kg converted to g) of all seafood and products were obtained from HM Revenue and Customs (HMRC) trade data. Average weekly per capita UK seafood purchases (g) were obtained from the Department for Environment, Food and Rural Affairs (DEFRA) family food data. UK seafood intake data were obtained from the National Dietary and Nutrition Survey (NDNS) (years 1 to 12). Seafood nutrient composition data were obtained from Department of Health and Social Care (DHSC), but complemented with data from Norwegian food composition tables for species where data from DHSC were lacking.*
Sampling strategy	*Data were retrieved from publicly available databases (see research sample).*
Data collection	*Data were retrieved by Anneli Lofstedt (first author) from publicly available databases*
Timing and spatial scale	*Data were retrieved between April 2022 and November 2023.*
Data exclusions	*We excluded data not intended for human consumption (e.g. farmed fish intended for coarse fishing, imported ornamental fish etc). Supplies were only calculated for species where production and trade data were provided. For Figures 3 and 4, only species with corresponding production, import, and export data were included (this equates to 30 species of a total of 73 species in the database).*
Reproducibility	*N/A*
Randomization	*N/A*
Blinding	*N/A*

Did the study involve field work? ☐ Yes ☒ No

Field work, collection and transport

Field conditions	*N/A*
Location	*N/A*
Access & import/export	*N/A*
Disturbance	*N/A*

Reporting for specific materials, systems and methods

We require information from authors about some types of materials, experimental systems and methods used in many studies. Here, indicate whether each material, system or method listed is relevant to your study. If you are not sure if a list item applies to your research, read the appropriate section before selecting a response.

Materials & experimental systems

n/a	Involved in the study
☒	☐ Antibodies
☒	☐ Eukaryotic cell lines
☒	☐ Palaeontology and archaeology
☒	☐ Animals and other organisms
☒	☐ Clinical data
☒	☐ Dual use research of concern
☒	☐ Plants

Methods

n/a	Involved in the study
☒	☐ ChIP-seq
☒	☐ Flow cytometry
☒	☐ MRI-based neuroimaging

Antibodies

Antibodies used: *N/A*

Validation: *N/A*

Eukaryotic cell lines

Policy information about cell lines and Sex and Gender in Research

Cell line source(s): *N.A*

Authentication: *N/A*

Mycoplasma contamination: *N/A*

Commonly misidentified lines (See ICLAC register): *N/A*

Palaeontology and Archaeology

Specimen provenance: *N/A*

Specimen deposition: *N/A*

Dating methods: *N/A*

☐ Tick this box to confirm that the raw and calibrated dates are available in the paper or in Supplementary Information.

Ethics oversight: *N/A*

Note that full information on the approval of the study protocol must also be provided in the manuscript.

Animals and other research organisms

Policy information about studies involving animals; ARRIVE guidelines recommended for reporting animal research, and Sex and Gender in Research

Laboratory animals: *N/A*

Wild animals: *N/A*

Reporting on sex: *N/A*

Field-collected samples: *N/A*

Ethics oversight: *N/A*

Note that full information on the approval of the study protocol must also be provided in the manuscript.

Clinical data

Policy information about clinical studies

All manuscripts should comply with the ICMJE guidelines for publication of clinical research and a completed CONSORT checklist must be included with all submissions.

Clinical trial registration	N/A
Study protocol	N/A
Data collection	N/A
Outcomes	N/A

Dual use research of concern

Policy information about dual use research of concern

Hazards

Could the accidental, deliberate or reckless misuse of agents or technologies generated in the work, or the application of information presented in the manuscript, pose a threat to:

No	Yes	
☒	☐	Public health
☒	☐	National security
☒	☐	Crops and/or livestock
☒	☐	Ecosystems
☒	☐	Any other significant area

Experiments of concern

Does the work involve any of these experiments of concern:

No	Yes	
☒	☐	Demonstrate how to render a vaccine ineffective
☒	☐	Confer resistance to therapeutically useful antibiotics or antiviral agents
☒	☐	Enhance the virulence of a pathogen or render a nonpathogen virulent
☒	☐	Increase transmissibility of a pathogen
☒	☐	Alter the host range of a pathogen
☒	☐	Enable evasion of diagnostic/detection modalities
☒	☐	Enable the weaponization of a biological agent or toxin
☒	☐	Any other potentially harmful combination of experiments and agents

Plants

Seed stocks	N/A
Novel plant genotypes	N/A
Authentication	N/A

ChIP-seq

Data deposition

- [ ] Confirm that both raw and final processed data have been deposited in a public database such as GEO.
- [ ] Confirm that you have deposited or provided access to graph files (e.g. BED files) for the called peaks.

Data access links<br>*May remain private before publication.*	*N/A*
Files in database submission	*N/A*
Genome browser session<br>(e.g. UCSC)	*N/A*

Methodology

Replicates	*N/A*
Sequencing depth	*N/A*
Antibodies	*N/A*
Peak calling parameters	*N/A*
Data quality	*N/A*
Software	*N/A*

Flow Cytometry

Plots

Confirm that:

- [ ] The axis labels state the marker and fluorochrome used (e.g. CD4-FITC).
- [ ] The axis scales are clearly visible. Include numbers along axes only for bottom left plot of group (a 'group' is an analysis of identical markers).
- [ ] All plots are contour plots with outliers or pseudocolor plots.
- [ ] A numerical value for number of cells or percentage (with statistics) is provided.

Methodology

Sample preparation	*N/A*
Instrument	*N/A*
Software	*N/A*
Cell population abundance	*N/A*
Gating strategy	*N/A*

- [ ] Tick this box to confirm that a figure exemplifying the gating strategy is provided in the Supplementary Information.

Magnetic resonance imaging

Experimental design

Design type	*N/A*
Design specifications	*N/A*
Behavioral performance measures	*N/A*

Acquisition

Imaging type(s) | N/A

Field strength | N/A

Sequence & imaging parameters | N/A

Area of acquisition | N/A

Diffusion MRI ☐ Used ☐ Not used

Preprocessing

Preprocessing software | *Provide detail on software version and revision number and on specific parameters (model/functions, brain extraction, segmentation, smoothing kernel size, etc.).*

Normalization | *If data were normalized/standardized, describe the approach(es): specify linear or non-linear and define image types used for transformation OR indicate that data were not normalized and explain rationale for lack of normalization.*

Normalization template | *Describe the template used for normalization/transformation, specifying subject space or group standardized space (e.g. original Talairach, MNI305, ICBM152) OR indicate that the data were not normalized.*

Noise and artifact removal | *Describe your procedure(s) for artifact and structured noise removal, specifying motion parameters, tissue signals and physiological signals (heart rate, respiration).*

Volume censoring | *Define your software and/or method and criteria for volume censoring, and state the extent of such censoring.*

Statistical modeling & inference

Model type and settings | N/A

Effect(s) tested | N/A

Specify type of analysis: ☐ Whole brain ☐ ROI-based ☐ Both

Statistic type for inference | N/A

(See Eklund et al. 2016)

Correction | N/A

Models & analysis

n/a	Involved in the study
☒	☐ Functional and/or effective connectivity
☒	☐ Graph analysis
☒	☐ Multivariate modeling or predictive analysis

Functional and/or effective connectivity | N/A

Graph analysis | N/A

Multivariate modeling and predictive analysis | N/A
